# Assessment of NLRP3 Gene Polymorphisms with Periodontitis as Compared with Healthy Periodontium in Iraqi Arabs Patients

**DOI:** 10.1055/s-0043-1761185

**Published:** 2023-02-22

**Authors:** Athraa A. Mahmood, Raghad Fadhil Abbas

**Affiliations:** 1 Department of Oral Surgery and Periodontology, College of Dentistry, Mustansiriyah University, Baghdad, Iraq; 2 Department of Periodontology, College of Dentistry, University of Baghdad, Baghdad, Iraq

**Keywords:** NLRP3, periodontitis, gene polymorphisms

## Abstract

**Objectives** The nod-like receptor pyrin domain-containing protein 3 (NLRP3) inflammasome regulates the maturation and release of the cytokines as well as the activation of caspase in response to danger signals derived from pathogenic infection, tissue damage, andmetabolic changes that have a role in the pathogenesis of different diseases as periodontitis. Yet, the susceptibility to this illness could be determined by population-based genetic differences. The aim of this study was to determine whether periodontitis in Arab populations from Iraq is correlated with NLRP3 gene polymorphisms and measure clinical periodontal parameters and investigate their association with genetic polymorphisms of the NLRP3.

**Materials and Methods** The study sample consisted of 94 participants ranging from 30 to 55 years old, both males and females who fulfilled the study's criteria. The selected participants were divided into two groups: the periodontitis group (62 subjects) and the healthy control group (32 subjects). The examination of clinical periodontal parameters of all participants was carried out, followed by a collection of venous blood for NLRP3 genetic analysis using the polymerase chain reaction–sequencing technique.

**Results** The genetic analysis of NLRP3 genotypes at four single nucleotide polymorphisms (SNPs) (rs10925024, rs4612666, rs34777555, and rs10754557), by Hardy–Weinberg equilibrium, identified nonsignificant differences in studied groups. The C-T genotype among periodontitis was significantly different from controls, while the C-C genotype among control was significantly different from periodontitis at NLRP3 rs10925024. Overall, there were 35 SNPs in the periodontitis group and 10 SNPs in the control group for rs10925024 with significant differences versus nonsignificant differences of the other SNPs between the studied groups. Clinical attachment loss and NLRP3 rs10925024 additionally demonstrated a significant positive correlation in the periodontitis subjects.

**Conclusion** The findings suggested that polymorphisms of the *NLRP3* gene may have a role and increasing the genetic susceptibility to periodontal disease in Arabs Iraqi patients.

## Introduction

Periodontitis is a complex multifactorial disease caused by several risk factors, including genetic risk factors that account for nearly half of the population's diversity in periodontitis risk. 1 Additionally, genetic factors account for the heterogeneity in disease location, severity, and dissemination in susceptible individuals. 2

Pathogenic microorganisms activate the host immune system. 3 4 Particularly, inflammasomes are intracellular pattern recognition receptors that become active when several signals are recognized as pathogen-associated molecular patterns (PAMPs). 5 The most intricate of them all, the nod-like receptor pyrin domain-containing protein 3 (NLRP3) inflammasomes react to PAMP. 6 Increased secretion of mature interleukin (IL)-1β, caspase-1, and IL-18, which have a critical role in the host innate immune reactions during periodontitis, is brought on by the NLRP3 inflammasomes' formation and activation. 6 7

Additionally, due to the influence of genetic polymorphisms, various ethnic groups may have different degrees of disease susceptibility. Some immunological genetic variations have been associated with periodontitis depending on the candidate gene approach and genome-wide association studies. 2 8 Many association studies with the candidate gene approach are not recommended as a standard technique to evaluate genetic involvement in periodontitis because they produce varying and frequently conflicting results due to a lack of statistical power and the absence of multiple testing corrections. Moreover, the phenotyping of periodontitis and control individuals has not been consistent among investigations. Furthermore, many studies have failed to account for lifestyle variables that may influence the periodontitis phenotype or the prevalence of comorbidities. 1 2 Thus, sufficient sample size, pilot studies, and inclusion and exclusion criteria in these studies are critical for determining genetic involvement.

The single nucleotide polymorphism (SNP) is the most common type of polymorphism, in which a single nucleotide is substituted by another nucleotide, especially those that are in the vicinity of protein-coding genes that able to affect or modify the corresponding gene expression or protein synthesis with influences on the periodontium's structural elements or the host's immunological reaction to a microbial attack. Polymorphisms can stimulate or retard the production of different cytokines. Furthermore, various studies have discovered that genotypic polymorphisms are connected with phenotypic variety, such as periodontitis severity and onset. 9 10 11 12 13 One such polymorphism is the NLRP3, there are a few studies that have looked at the NLRP3 polymorphism as it relates to periodontitis. 14 Hence, the purpose of this study was to explore the association of polymorphisms in the *NLRP3* gene with periodontitis in the Arabs populations of Iraq.

## Materials and Methods

### Patient Selection

The design of our study was an observational case–control study. Each participant received thorough explanations of the study and its methods, and their informed consent was obtained using a form that had been approved by the College of Dentistry, University of Baghdad ethics committee, reference number: 453.

The potential patients were recruited consecutively from the attendants to the teaching hospital of the College of Dentistry, Baghdad University and the Iraqi National Centre for blood donation during the period from March to August 2022.

The study included similar ethnic backgrounds of Iraqi Arabs subjects, systemically healthy males or females without over- or underweight and height (18.5–29.9) according to body mass index 15 with the presence of at least 20 or more natural teeth, willingness to sign a form of informed consent, no antibiotics therapy has been received in the last 3 months, and during the last 6 months, there was no periodontal therapy. Any pregnant, lactating women and individuals with dental implants and orthodontic or prosthodontic appliances were excluded.

The selected participants were classified into two groups based on their periodontal clinical conditions. The control group with healthy intact periodontium, who have no gingival inflammation signs at all “bleeding on probing (BOP) <10%, probing pocket depth (PPD) ≤ 3 mm, and no probing or clinical attachment loss (CAL).” 16 In addition, the periodontitis group, which is defined, following the 2017 classiﬁcation of periodontal diseases and conditions, as the interdental CAL is identified at ≥ 2 teeth (not adjacent), or oral CAL ≥ 3 mm and PPD > 3 mm is identified at ≥ 2 teeth. 17 Periodontitis causes must exhibit unstable status (PPD ≥ 5 mm or PPD 4 mm with BOP) with no risk factors (diabetes mellitus [DM] and/or smoking).

### Pilot Study and Sample Size

A pilot study was conducted using the first samples collected from each group following an allocation ratio of 1:2 (periodontal health:periodontitis, respectively) (three periodontal health and six periodontitis). A total of nine samples were analyzed in the laboratory by polymerase chain reaction (PCR)-sequencing methods to validate the primers and if any preparation was required for blood samples. The values obtained from this pilot study were then added to the final data of the main study.

The sample size was determined from the following equation 18 :

Sample size = ( *r* + 1)/ *r* × *P* (1 − *P* ) ( *Z*_1 − β_ + *Z*_1 − α/2_ ) ^2^ /( *P* 1 − *P* 2) ^2^

where *r* is the ratio of cases to controls equal to 2; *P* is the proportion of population equal to ( *P* 1 + *P* 2)/2; *Z*_1 − β_ is the desired power (0.84 for 80% power); *Z*_1 − α/2_ is the statistical value that corresponds to the level of confidence (95%) that is equal to (1.96); *P* 1 is the proportion in cases, which was estimated at 66% of genetic polymorphisms of the NLRP3 at rs10925024 in the pilot study; and *P* 2 is the proportion in controls, which was estimated at 33% of genetic polymorphisms of the NLRP3 at rs10925024 in the pilot study. Then, after substituting the values into the above equation:

Sample size = (2 + 1)/2 × 0.5 (1 − 0.5) (0.84 + 1.96) ^2^ /(0.66 − 0.33) ^2^

Sample size = 26.98 = 27 + 3 (taking into account the 10% dropout rate of participants). Thus, the total sample size for the groups must be 90 at a minimum (60 for cases and 30 for control with an allocation ratio of 2:1).

### Clinical Examination

Before conducting any periodontal examination and the collection of blood samples, every precaution needed to avoid coronavirus disease 2019 infection was followed. Then, the clinical parameters to be measured in logic sequence included (plaque index [PI, BOP, PPD, CAL, gingival recession indices, and the number of missing teeth) except the third molars. PI and BOP were identified as present (1) or absent (0). 19 20 The periodontal examination was achieved by using a periodontal probe (Michigan 0 probe) at six sites/teeth except for plaque scores of only four surfaces for all teeth.

The accuracy, validity, and reproducibility of the researcher were assessed by carrying out an inter- and intraexaminer calibration; categorical variables (PI and BOP) were assessed by using a kappa-coefficient assay. The targeted level was kappa value ≥75% to decide a good level of agreement was present. For continuous variables (PPD and CAL), the level of agreement rounded to the nearest millimeter should be >0.9 as determined by interclass coefficient assay.

### Sample Collection and DNA Extraction

Each individual had a venipuncture performed using a 5-mL disposable syringe and a 20-gauge needle to obtain 2 mL of blood from the antecubital fossa under an aseptic technique with 70% ethanol alcohol. The collected blood was placed into ethylenediaminetetraacetic acid tube (1.5 mg/mL) and stored at −40°C for the (NLRP3) genetic analysis by PCR-sequencing methods. The processes include deoxyribonucleic acid (DNA) extraction, PCR amplification, sequencing, and data analysis.

During the first step of the study, blood samples were used to isolate genomic DNA following the instructions provided by the ABIOpure extraction kit (United States), then the extracted DNA was quantified by Quantus Fluorometer (Promega, United States).

### Polymorphism Detection

In detail, primer preparation and optimization were the first step in the PCR amplification process. The primers in this study used for the detection of the *NLRP3* gene were designed by PrimerQuest from Integrated DNA Technology. The primers covered the majority of the gene to identify any potential alterations that might have been present in the study groups and to compare them to typical sequences. The Macrogen Company provided these primers in lyophilized form as a forward primer (5-TGTAAAACGACGGCCAGTCTAGACTCGAAAGGGCAAATAC-3) and reverse primer (5-CAGGAAACAGCTATGACCTGCACCATCTCTAAGGTTTC-3) with 964 product size (bp) at 60 annealing temperatures.

To prepare primer stock solution, lyophilized primers were diluted in nuclease-free water (NFW) to a total of 100 pmol/μL for both reverse and forward tubes followed by vertexing. Then, a primer working solution containing 10 pmol/μL was prepared by adding 10 µL of stock solution and 90 µL of NFW for reverse primer and forward primer in a 1.5-mL tube (stored at freezer −40°C).

To assess the ideal annealing temperature for primers, the DNA sample was replicated with the same primers at treatment temperatures of 55, 58, 60, 63, and 65°C. After that, the PCR amplifications were completed. The PCR cycling procedure was carried out by PCR express (Thermal Cycler, Bio-Rad, United States) with the following scheme, denatured at 94°C/4 minutes, then 30 denaturation cycles at 94°C/30 seconds, followed by annealing at 55, 58, 60, 63, or 65°C/30 seconds, and extension at 72°C/30 seconds. Following a final extension incubation at 72°C/7 minutes, the processes were stopped by incubating for 10 minutes at 4°C.

**Fig. 1:**
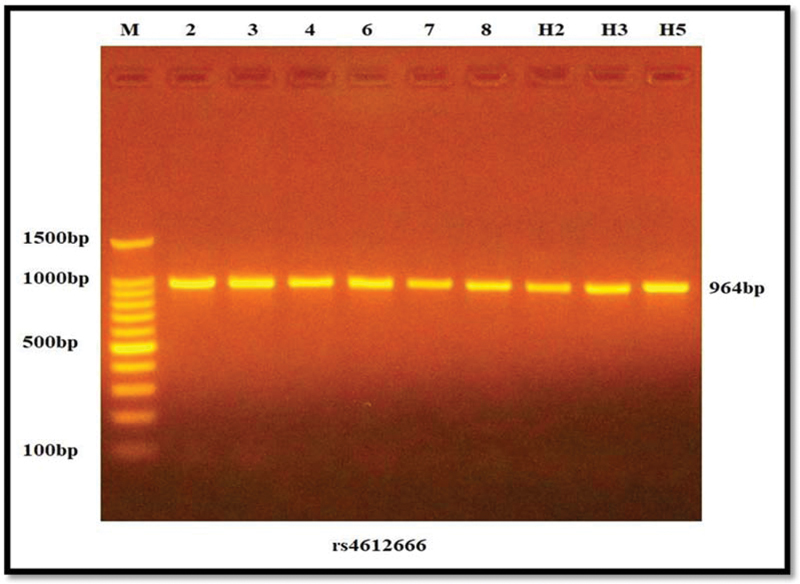
Electrophoresis of the amplification of the *NLRP3* gene at 964 bp for some samples. M, 100bp DNA ladder marker.

Agarose gel electrophoresis (OWL, Thermo, United States) supplemented with 10 mg/mL ethidium bromide staining (EBS) (Promega, United States) was used to verify the existence of amplification following PCR amplification. Then, 5 μL of the PCR cycling product was added straight to the well; 100 volts and 50 amps of electricity were turned on for 60 minutes. From the negative (cathode) to the positive (anode) poles, DNA travels. Under ultraviolet light, EBS bands in the gel were viewed, and then gel imaging equipment was used to take a photograph (Major Science, Taiwan) ( Fig. 1 ).

Then, PCR products were sent to Macrogen Company, Korea for Sanger sequencing using ABI3730XL, automated DNA sequences, after the gene's fragments appear in the PCR with the anticipated size to identify the nucleotide sequences of these fragments. The nucleotide sequences of the samples were received by e-mail and compared with the source nucleotide sequence and then analyzed by the software program Basic Local Alignment Search Tool (BLAST).

## Statistical Methods' Outcomes

Data description and analysis were achieved by Statistical Package for Social Science (SPSS version 28, IBM, United States). Mean, standard deviation, and median were used for the continuous data, while frequency (number) and percentage were used for categorical variables.

The normality of distribution was checked by the Shapiro–Wilk and D'agostino's tests. Mann–Whitney's *U* tests determined the significant difference between the examined groups. In addition, categorical variables were analyzed by using the chi-square test. The correlation between clinical parameters and NLRP3 polymorphisms genes was identified by Spearman's correlation test. The significant difference will be revealed if *p* < 0.05.

The intergroup differences in the distributions of the SNPs were analyzed using the chi-square test. Frequency distributions for selected variables were done first. Hardy–Weinberg equilibrium (HWE) is used to calculate the expected alleles from the observed genotypes. The odds ratio (OR) measures the strength of association between the presence of certain polymorphisms and disease status.

## Results

A total of 94 subjects were enrolled out of the 940 subjects whose eligibility was determined. In brief, the average age was 41.66 ± 8.176 (median 40.5) and 38.66 ± 6.553 (median 37) with a range between 30 and 55 years for the periodontitis and control groups, respectively. In addition, distribution according to sex and family history is summarized in Table 1 .

**Table 1:** Statistics of demographic and clinical parameters of the studied population

Parameters		Healthy (32)	Periodontitis (62)	*p* -Value
**Age**	Mean ± SD	38.66 ± 6.553	41.66 ± 8.176	0.091 NS a
	Median	37	40.50	
**Sex** b	Male	19 (59.4%)	37 (59.7%)	0.977 NS
	Female	13 (40.6%)	25 (40.3%)	
**Family history** b	Positive	2 (6.3%)	10 (16.1%)	0.174 NS a
	Negative	30 (93.8%)	52 (83.9%)	
**PI**	Mean ± SD	0.156 ± 0.100	0.634 ± 0.223	0.000 c
	Median	0.122	0.676	
**BOP**	Mean ± SD	0.055 ± 0.032	0.472 ± 0.180	0.000 c
	Median	0.056	0.450	
**PPD**	Mean ± SD	0.000 ± 0.000	4.524 ± 0.614	0.000 c
	Median	0.000	4.3150	
**CAL**	Mean ± SD	0.000 ± 0.000	3.458 ± 0.992	0.000 c
	Median	0.000	3.300	
**GR**	Mean ± SD	0.000 ± 0.000	2.230 ± 1.153	0.000 c
	Median	0.000	2.230	
**Teeth** b	Lost teeth	16 (1.8%)	198 (11.4%)	0.000 d
	Mobile teeth	0 (0.0%)	46 (2.6%)	
	Present teeth	880 (98.2%)	1,538 (85.9%)	

Abbreviations: BOP, bleeding on probing, CAL, clinical attachment loss, GR, gingival recession; NS, nonsignificant; PI, plaque index, PPD, probing pocket depth, SD, standard deviation.

a Nonsignificant at *p* ≥ 0.05 by Mann–Whitney's test and for the chi-square test.

b Number (%).

c Significant at *p* ≤ 0.001 by Mann–Whitney's test.

d Significant at *p* ≤ 0.001 for the chi-square test.

Regarding the result of the clinical examination, the periodontitis subjects had significantly greater PI, BOP, and a greater number of missing and mobile teeth at *p* < 0.001 ( Table 1 ). As well, the mean of PPD in the periodontitis group was 5.32 ± 0.687 and the mean of CAL was 6.52 ± 0.667, whereas the control group was free from PPD and CAL.

**Fig. 2:**
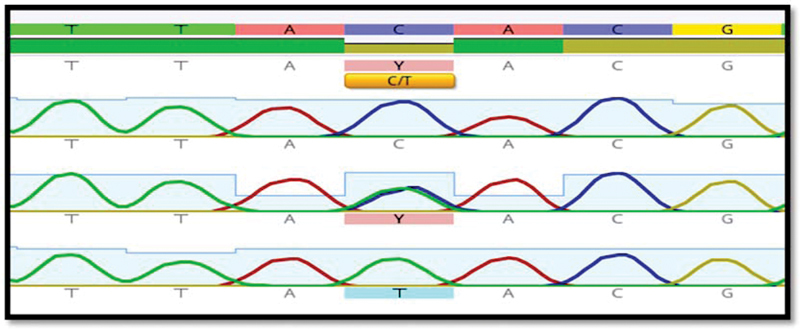
*NLRP3* gene's rs10925024 SNP analysis by Sanger sequencing. A homozygous “C” allele is indicated by a single “C” peak. Homozygous T allele indicated by a single “T” peak. The “C” and “T” peaks are a sign of the heterozygous C/T allele. SNP, single nucleotide polymorphism.

**Fig. 3:**
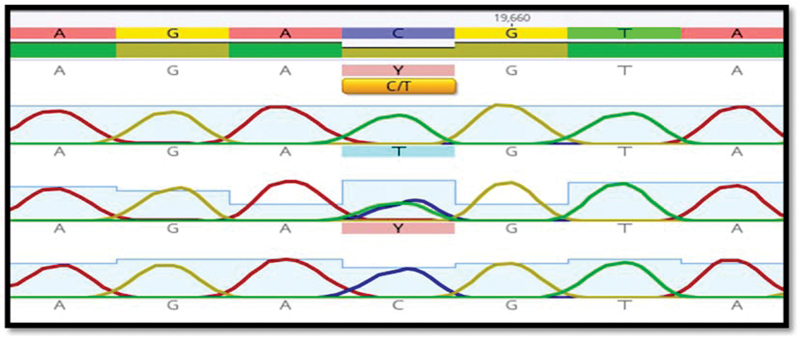
Sanger sequencing analysis of the *NLRP3* gene's rs4612666 SNP. Homozygous T allele denoted by a single “T” peak. A homozygous “C” allele is denoted by a single “C” peak. The “T” and “C” peaks are a sign of the T/C heterozygous allele. SNP, single nucleotide polymorphism.

**Fig. 4:**
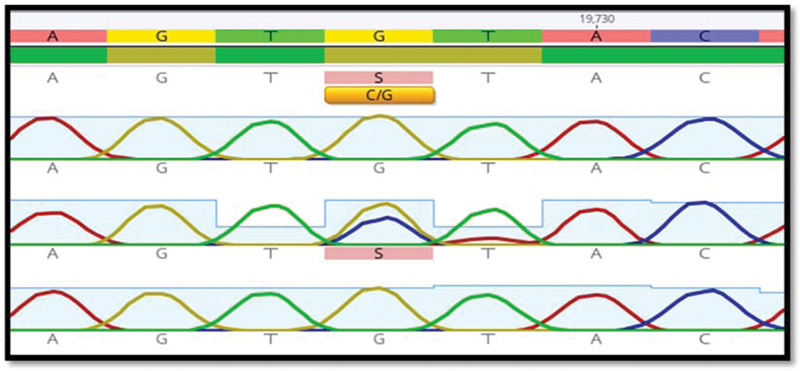
Sanger sequencing analysis of the *NLRP3* gene's rs34777555 SNP. The G homozygous allele is denoted by a single “G” peak. A homozygous “C” allele is denoted by a single “C” peak. The “G” and “C” peaks are a sign of the G/C heterozygous allele. SNP, single nucleotide polymorphism.

**Fig. 5:**
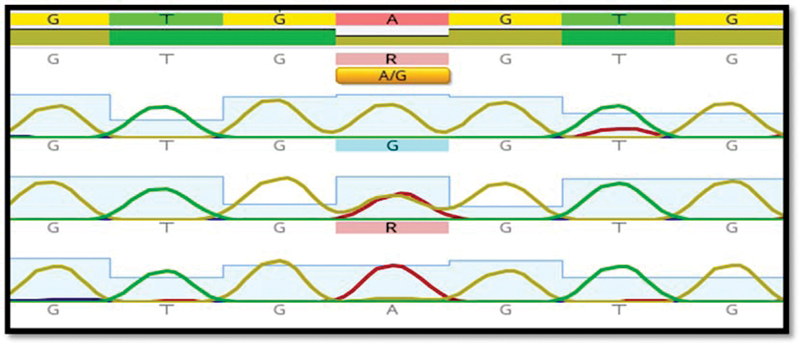
Sanger sequencing analysis of the *NLRP3* gene's rs10754557 SNP. The G homozygous allele is shown by a single “G” peak. A homozygous allele is shown by a single “A” peak. The “G” and “A” peaks are a sign of the G/A heterozygous allele. SNP, single nucleotide polymorphism.

Furthermore, the sequencing of NLRP3 SNPs by the Sanger method was described for some samples in Figs. 2 to 5 for rs10925024, rs4612666, rs34777555, and rs10754557, respectively, and there was the substitution of allele cytosine (C) with thymine (T) in rs10925024 SNP, while there was the substitution of allele T with C in rs4612666 SNP. In addition, there was a substitution of allele guanine (G) with C in rs34777555 SNP and a substitution of allele G with adenine (A) in rs10754557 SNP.

Analysis by HWE for studied groups was done for the NLRP3 SNPs to compare the observed genotype with the expected genotype in each studied group as well as total samples. The equilibrium was nonsignificant, as shown in Table 2 .

**Table 2:** The HWE for studied groups in NLRP3 polymorphism

NLRP3		All groups		Healthy		Periodontitis	
rs	Genotype	Observed	Expected	Observed	Expected	Observed	Expected
10925024	C-C	49	49.2	22	21.1	27	28.5
	C-T	38	37.6	8	9.8	30	27.1
	T-T	7	7.2	2	1.1	5	6.5
	HWE	0.009		1.030		0.711	
	*p* -Value	0.921 NS		0.309 NS		0.398 NS	
4612666	T-T	7	6.9	3	3.4	4	3.6
	T-C	37	37.2	15	14.1	22	22.7
	C-C	50	49.9	14	14.4	36	35.6
	HWE	0.0018		0.127		0.065	
	*p* -Value	0.965 NS		0.721 NS		0.797 NS	
34777555	G-G	91	91.0	31	31.0	60	60.0
	G-C	3	3.0	1	1.0	2	2.0
	C-C	0	0.0	0	0.0	0	0.0
	HWE	0.024		0.008		0.016	
	*p* -Value	0.875 NS		0.928 NS		0.897 NS	
10754557	G-G	11	11.9	4	5.3	7	6.8
	G-A	45	43.1	18	15.4	27	27.4
	A-A	38	38.9	10	11.3	28	27.8
	HWE	0.178		0.881		0.016	
	*p* -Value	0.672 NS		0.347 NS		0.898 NS	

Abbreviations: A, adenine; C, cytosine; G, guanine; HWE, Hardy–Weinberg equilibrium; NLRP3, nod-like receptor pyrin domain-containing protein 3; NS, *p* ≥ 0.05 nonsignificant; rs, reference single nucleotide polymorphism; T, thymine.

Concerning this, the distribution of NLRP3 SNPs according to their types in the studied groups' samples is illustrated in Table 3 . All the detected SNPs after sequencing were of single base substitution, 148 (98.7%) of them were transition and 2 (1.3%) transversion SNPs in the periodontitis, while in the healthy, 67 (98.5%) were transition and 1 (1.5%) transversion SNPs. The transition mutations belong to rs10925024, rs4612666, and rs10754557, whereas transversion mutations belong to rs34777555. There was a nonsignificant difference ( *p* < 0.05) between the studied groups in SNPs type distribution.

**Table 3:** The types of NLRP3 SNPs in study groups

Types of SNPs	Healthy a		Periodontitis a		X ^2^	*p* -Value
Transition	67	98.5%	148	98.7%	0.006	0.936 NS
Transversion	1	1.5%	2	1.3%		
Total	3	1.4%	215	98.6%		

Abbreviations: NLRP3, nod-like receptor pyrin domain-containing protein 3; NS, *p* ≥ 0.05 nonsignificant; SNPs, single nucleotide polymorphisms; X ^2^ , chi-square.

a Number (%).

The SNPs number and distribution in both groups are illustrated in Table 4 , as there were 35 SNPs in the periodontitis group for rs10925024 and 10 SNPs in the control group for the same rs with significant differences.

**Table 4:** The number of SNPs for NLRP3 in periodontitis patients and control samples

NLRP3	Healthy a		Periodontitis a		OR (95% CI)	X ^2^	*p* -Value
rs10925024	10	31.3%	35	56.5%	2.852 (1.159–7.018)	5.372	0.020 b
rs4612666	29	90.6%	58	93.5%	1.500 (0.315–7.152)	0.262	0.609 NS
rs34777555	1	3.1%	2	3.2%	1.033 (0.090–11.847)	0.001	0.979 NS
rs10754557	28	87.5%	55	88.7%	1.122 (0.303–4.160)	0.030	0.863 NS

Abbreviations: CI, confidence interval; NLRP3, nod-like receptor pyrin domain-containing protein 3; NS, *p* ≥ 0.05 nonsignificant; OR, odds ratio; SNPs, single nucleotide polymorphisms; X ^2^ , chi-square.

a Number (%).

b Significance level at *p* < 0.05.

While there were 58 SNPs in the periodontitis group and 29 SNPs in the healthy group at rs4612666 with a nonsignificant difference between them. On the other hand, there were two SNPs in the periodontitis and one SNP in the healthy group at rs34777555 with a nonsignificant difference between them. In addition, there were 55 SNPs in the periodontitis and 28 SNPs in the healthy group at rs10754557 with a nonsignificant difference between them.

Interestingly, the effect of NLRP3 SNPs on periodontitis distribution was assessed by counting OR and it was found 2.852, 1.500, 1.033, and 1.122 for rs10925024, rs4612666, rs34777555, and rs10754557, respectively.

The genetic analysis for NLRP3 SNPs in the present study revealed the genotype and allele frequency in the studied groups as shown in Table 5 , the chi-square test showed a nonsignificant difference in their distribution between studied groups at *p* -value ≥ 0.5 for all SNPs, except for the C-C and C-T genotypes of rs10925024 showed a significant difference. In a more detailed description of the effect of genotype on disease distribution, the study illustrates the effect of each genotype of NLRP3 SNPs on disease distribution. It was found that genotype C-T had a higher OR (2.813) than other genotypes to develop the disease in rs10925024. While the genotype T-T has a 1.378 chance to develop the disease in comparison with the other genotypes located in the rs10925024.

**Table 5:** Analytic statistics of the genotype and allele distribution of NLRP3 polymorphism

NLRP3	Genotype/allele	Healthy a		Periodontitis a		OR (95% CI)	X ^2^	*p* -Value	RR
rs10925024	C-C	22	68.8%	27	43.5%	0.351 (0.143–0.863)	5.372	0.020 a	0.633
	C-T	8	25%	30	48.4%	2.813 (1.096–7.218)	4.793	0.029 a	1.935
	T-T	2	6.3%	5	8.1%	1.316 (0.241–7.191)	0.101	0.751 NS	1.290
	C	52	81.3%	84	67.7%	0.485 (0.233–1.008)	3.849	0.050 NS	0.834
	T	12	18.8%	40	32.3%	2.063 (0.992–4.290)	3.849	0.050 NS	1.720
rs4612666	T-T	3	9.4%	4	6.5%	0.667 (0.140–3.179)	0.262	0.609 NS	0.688
	T-C	15	46.9%	22	35.5%	0.623 (0.262–1.484)	1.147	0.284 NS	0.757
	C-C	14	43.8%	36	58.1%	1.780 (0.752–4.213)	1.737	0.188 NS	1.327
	T	21	32.8%	30	24.2%	0.653 (0.336–1.270)	1.586	0.208 NS	0.737
	C	43	67.2%	94	75.8%	1.530 (0.788–2.973)	1.586	0.208 NS	1.128
rs34777555	G-G	31	96.9%	60	96.8%	0.968 (0.084–11.09)	0.001	0.979 NS	0.999
	G-C	1	3.1%	2	3.2%	1.033 (0.09–11.847)	0.001	0.979 NS	1.032
	C-C	0	0.0%	0	0.0%	–	–	–	–
	G	63	98.4%	122	98.4%	0.968 (0.086–10.88)	0.001	0.979 NS	0.999
	C	1	1.6%	2	1.6%	1.033 (0.092–11.61)	0.001	0.979 NS	1.032
rs10754557	G-G	4	12.5%	7	11.3%	0.891 (0.240–3.302)	0.030	0.863 NS	0.903
	G-A	18	56.3%	27	43.5%	0.600 (0.254–1.418)	1.365	0.243 NS	0.774
	A-A	10	31.3%	28	45.2%	1.812 (0.737–4.454)	1.696	0.193 NS	1.445
	G	26	40.6%	41	33.1%	0.722 (0.387–1.347)	1.052	0.305 NS	0.814
	A	38	59.4%	83	66.9%	1.385 (0.742–2.584)	1.052	0.305 NS	1.127

Abbreviations: A, adenine; C, cytosine; CI, confidence interval; G, guanine; NLRP3, nod-like receptor pyrin domain-containing protein 3; NS, *p* ≥ 0.05 nonsignificant; OR, odds ratio; RR, relative reduction; T, thymine; X ^2^ , chi-square.

a Number (%).

a Significance level at *p* < 0.05.

Moreover, for rs10925024, there was a high frequency of the C allele in the healthy group than in the periodontitis, while the periodontitis showed a higher T allele than that in the healthy, conversely concerning rs4612666. On the other hand, the frequency of the A allele was higher in the periodontitis group than that in the healthy, while the healthy showed a higher G allele than that in the periodontitis for rs10754557. Finally, the frequency of G and C alleles in the control and periodontitis groups for rs34777555 was equal.

Surprisingly, the present study found the T allele and C-T genotype association with periodontitis (OR = 2.063, confidence interval [CI] = 0.992–4.290, and relative reduction [RR] = 1.720 and OR = 2.813, CI = 1.096–7.218, and RR = 1.935, respectively) for rs10925024.

The analytic statistic in this study found a nonsignificant association of NLRP3 SNPs with demographic and clinical parameters in both periodontitis and control groups at a *p* -value ≥ 0.05. However, there was a significant (positive) correlation between rs10925024 and CAL, as shown in Table 6 .

**Table 6:** Association of NLRP3 SNPs with demographic and clinical characteristics

Parameters	rs10925024		rs4612666		rs34777555		rs10754557	
	*r*	*p* -Value	*r*	*p* -value	*r*	*p* -Value	*r*	*p* -Value
Age	0.089	0.396 NS	0.036	0.729 NS	0.054	0.607 NS	0.122	0.242 NS
Tooth loss	0.111	0.287 NS	0.100	0.337 NS	0.027	0.793 NS	0.164	0.115 NS
Tooth mobile	0.030	0.773 NS	0.089	0.393 NS	0.088	0.400 NS	0.132	0.205 NS
Sex	−0.023	0.827 NS	0.047	0.654 NS	–0.150	0.150 NS	–0.186	0.073 NS
Family history	0.162	0.118 NS	0.090	0.388 NS	0.069	0.506 NS	0.023	0.828 NS
PI	0.109	0.298 NS	0.088	0.400 NS	0.081	0.435 NS	0.201	0.052 NS
BOP	0.160	0.124 NS	0.115	0.269 NS	0.001	0.991 NS	0.031	0.767 NS
PPD	0.176	0.090 NS	0.016	0.880 NS	–0.016	0.878 NS	0.039	0.706 NS
CAL	0.206	0.046 a	0.063	0.546 NS	0.047	0.655 NS	0.002	0.985 NS
GR	0.143	0.168 NS	0.123	0.238 NS	–0.075	0.471 NS	0.054	0.603 NS

Abbreviations: BOP, bleeding on probing; CAL, clinical attachment loss; GR, gingival recession; NLRP3, nod-like receptor pyrin domain-containing protein 3; NS, *p* ≥ 0.05 nonsignificant; PI, plaque index; PPD, probing pocket depth; *r,* Spearman's rank correlation; SNPs, single nucleotide polymorphisms.

a Significance level at *p* < 0.05.

Concerning the correlation among SNPs of NLRP3, the result showed a nonsignificant association at a *p* -value ≥0.05 in the studied groups between rs10925024 and rs34777555 as well as between rs10754557 and rs34777555. While there was a significant correlation among other SNPs of NLRP3, as illustrated in Table 7 .

**Table 7:** Correlation of NLRP3 SNPs with each other

NLRP3		*r*	*p* -Value
rs10925024	rs4612666	0.332	0.001 a
	rs34777555	−0.060	0.565 NS
	rs10754557	0.228	0.027 b
rs4612666	rs34777555	−0.217	0.035 b
	rs10754557	0.374	0.000 c
rs34777555	rs10754557	−0.066	0.525 NS

Abbreviations: NLRP3, nod-like receptor pyrin domain-containing protein 3; NS, *p* ≥ 0.05 nonsignificant; *r* , Spearman's rank correlation; SNPs, single nucleotide polymorphisms.

a Significance level at *p* ≤ 0.01.

b Significance level at *p* < 0.05.

c Significance level at *p* ≤ 0.001.

## Discussion

In the present investigation, NLRP3 polymorphisms were discovered that may indicate higher genetic susceptibility to periodontitis. This was supported by findings that the NLRP3 rs10925024 was positively correlated with elevated CAL. The current study, to the authors' knowledge, is the first study to investigate the relationship between genetic variation of NLRP3 and periodontitis risk in an Iraqi Arab population.

Multiple variables, including genetics, influence the progression of periodontitis, which is a complex disease. 21 22 As a potential pathogenic factor for periodontal disease, SNP of cytokines and inflammatory mediators have drawn more attention in recent research. 14 23

This research may contribute to the development of innovative treatment and preventive approaches as well as a better understanding of the pathophysiology of periodontitis. 24 Pathogenic bacteria in the tooth biofilm are responsible for the onset and progression of periodontitis because they induce an inflammatory immune reaction that causes tissue damage in a susceptible host. 25 26 The pathogenic periodontal pockets biofilm in conjunction with the host's immune-inflammatory reactions is known to interact in various ways to cause periodontal disease. Members of the inflammasome family including NLRP3 have a crucial role in immune responses in periodontitis, which results in elevated IL-1β and IL-18 secretion. 6 The role of NLRP3 polymorphism and greater genetic susceptibility to periodontitis revealed contradictions and highlighted a knowledge gap in this field. 14 27

Advances for studying periodontal inflammation have emerged with the development of the inflammasome concept. Polymorphism of NLRP3 is one of the most studied SNPs of all the inflammasomes in inflammatory and autoimmune diseases; NLRP3 has been implicated as having a key role in the pathogenesis of several diseases in the general population, including obesity, DM, rheumatoid arthritis, 28 29 all of which are clinical diseases known for their association with periodontitis. 14 23 30 31

In our study, we detected a significant difference between the examined groups of Iraqi subjects concerning the SNPs at position rs10925024 of NLRP3. As a result, 35 SNPs were found in the periodontitis cohort, while 10 SNPs were found in the healthy. Thus, the current finding revealed a significantly higher frequency of CT + TT genotypes of NLRP3 at rs10925024 was found in the periodontitis group accounting for 56.5% in contrast to 31.3% in healthy controls and is associated with an increased likelihood of having periodontitis compared with those with the CC genotype by (OR = 2.852) times. On the other hand, allele frequencies in study groups appear to be more frequent in the periodontitis group 32.3% versus 18.8% in controls. The allele and genotype frequencies complied with HWE. Unfortunately, there are no available references to compare the result of NLRP3 rs10925024 with it.

The CT genotype at rs10925024 was significantly higher in patients with periodontitis with a percentage of 48.4% as compared with 25% in the healthy. In addition, we observed a remarkable enhancement in the proportion of the T allele in rs10925024 of periodontitis, whereas there was a significant drop in the proportion of the C allele at the same location. This, in turn, increases the transcriptional activity of the *NLRP3* gene, and overexpression of cytokine and caspase, which leads to an exacerbated inflammation pathway in the periodontal tissue during the pathogenesis of the periodontal disease and leads to bone loss. 14 27 32 This is corroborated by the significant association between NLRP3 polymorphism and rising attachment loss.

Thus, it is biologically plausible that individuals with NLRP3 polymorphisms have increased genetic susceptibility to periodontitis due to increased cytokine production after NLRP3 activation. 32 The disparities in outcomes among various races or particular ethnicities, however, might be explained by the presence of distinct alleles responsible for susceptibility to the same disease.

While there was a slightly higher frequency of TC + CC genotypes of NLRP3 at rs4612666 in periodontitis which accounts for 93.5 versus 90.6% in healthy controls and is associated with an increased likelihood of having periodontitis compared with those with the TT genotype (OR = 1.500) times. On the other hand, the frequency of the genotypes (GC + CC) was higher in the periodontitis 3.2% than in the healthy group 3.1% at rs34777555 with a nonsignificant difference between them. In addition, there was a slightly higher frequency of GA + AA genotypes at rs10754557 in the periodontitis 88.7% than in the healthy group 87.5% with a nonsignificant difference between them. Thus, this study found no association between the NLRP3 polymorphism at positions rs4612666, rs34777555, and rs10754557 with periodontitis. In this respect, some studies have also found no relationship between these NLRP3 polymorphisms and inflammatory conditions, 33 34 although Isaza-Guzmán et al reported an association between NLRP3 rs4612666 and chronic periodontitis in the Colombian population 35 ; however, this study was lacking the ethnicity of chronic periodontitis patients and healthy controls, which may have explained some confounding influence of population stratification that could not be detected.

The results of a study on a Brazilian population indicated the reverse pattern, with a greater frequency of the T-C genotype in periodontitis patients. These findings were in contrast to those of this study. 32

The inconsistent findings in the literature may be attributed to sampling size, racial differences, case definitions of studied groups (periodontal disease and health), and different environmental and pathological factors that influence the host response. As a result, the proportion of polymorphism in a particular genotype may vary between populations or racial groups.

Men have been thought to be more prone to periodontal disease than women because of hormonal variables, poor personal hygiene practices, and poor health prevention behaviors, according to various research. 36 In addition, age is one of the confounding factors for periodontitis 37 ; thus, studied groups were matched with a nonsignificant difference in age and gender.

There were some limitations with the current study, it is important to investigate other confounding factors such as environmental and pathological that may contribute to or amplify a specific genetic variant to understand how genetics affect complex diseases. Just for uniformity and to avoid bias, other periodontal disease risk variables such as smoking, diabetes, and obesity were excluded from this study. Despite this, they may have an impact on the susceptibility to periodontitis in the presence of different polymorphisms. The genetic heterogeneity of periodontitis is an additional limitation that prevents generalizing the findings. Moreover, observational study results only provide an association, not causality, which must be further evaluated through higher evidence-based trials. Yet, this research was one of the few to look into the genetic basis of periodontitis in the Iraqi Arabs community with sufficient sample size and pilot study to allow for multiple testing corrections, which prevented many false-positive results associated with candidate gene approach studies.

Future trends are required to identify the association of periodontitis with genetic polymorphisms of other influential mediators (inflammation-associated molecules) such as ILs and the Fc receptor, especially in a sufficient sample size through higher evidence-based trials.

## Conclusion

The present study concluded that NLRP3 SNP may have a role in periodontitis genetic susceptibility in the population. Furthermore, the positive association between CAL and rs10925024 NLRP3 polymorphisms suggested a possible role in increasing the severity of periodontal disease. The discovery of genetic markers for periodontitis susceptibility will enable early detection of those at high risk and may ultimately aid through personalized types of therapy. More research will be needed to evaluate NLRP3's contribution to periodontitis in addition to other genetic, pathological, and environmental risk factors because of its complicated regulatory system.
